#  (−)-Epigallocatechingallate induces apoptosis in B lymphoma cells via caspase-dependent pathway and Bcl-2 family protein modulation

**DOI:** 10.3892/ijo.2015.2869

**Published:** 2015-02-03

**Authors:** JIANGYAN WANG, YU’AN XIE, YAN FENG, LITU ZHANG, XINPING HUANG, XIAOYUN SHEN, XIAOLING LUO

**Affiliations:** Research Department, Affiliated Tumour Hospital of Guangxi Medical University, Nanning 530021, P.R. China

**Keywords:** (−)-epigallocatechingallate, B lymphoma, apoptosis, caspase, pathway

## Abstract

(−)-Epigallocatechingallate (EGCG) as a representative polyphenol has attracted increasing attention due to its diversified effects, especially its potential as an agent for the prevention or treatment of certain cancers. However, the molecular mechanisms of EGCG-induced apoptosis in B lymphoma cells are unclear. The aim of this study was to investigate the effect of EGCG on proliferation and apoptosis in the B lymphoma cell lines Jeko-1 and Raji, and determine the underlying mechanisms. Cell proliferation and cytotoxicity were determined by the cell counting kit (CCK-8) assay; apoptosis was assessed by flow cytometry using the Annexin V-PE/7AAD double staining; Fas, Bcl-2 and Bax mRNA expression levels were determined by real-time PCR; caspase activity was measured by the caspase activity assay kit; the expression levels of apoptosis-associated proteins were determined by western blot analysis. We demonstrated that EGCG induced growth inhibition and apoptosis in a dose- and time-dependent manner. In agreement, EGCG upregulated the mRNA expression of Fas and Bax while downregulating Bcl-2. Protein expression levels of Bax, activated caspase-3, -7, -8, and -9, and PARP were increased, while Bcl-2 protein levels were reduced by EGCG treatment. Taken together, EGCG induces B lymphoma cell apoptosis by triggering caspase-dependent intrinsic (mitochondrial) and extrinsic (death receptor) pathways. These findings suggest that EGCG may be a potential agent for the treatment of B lymphoma.

## Introduction

B cell lymphoma (BCL) includes Mantle cell lymphoma (MCL), primary effusion lymphoma (PEL), Burkitt’s lymphoma (BL) and diffuse large B-cell lymphoma (DLBCL) (1); both MCL and BL are highly aggressive non-Hodgkin lymphomas (NHLs). MCL represents ~4% of all lymphomas in the US and 7–9% in Europe, with a median overall survival (OS) of 4–5 years and poor susceptibility to standard chemotherapy strategy (2,3). BL constitutes ~1–2% of all adult NHL cases and 30–50% of all childhood lymphomas in Western countries (4). The current chemotherapy regimens for MCL and BL are not very effective and a number of patients cannot afford the dose-intensive side effects, although dose-intensive induction regimens result in the improvement of median OS (5). Therefore, finding new therapy options is urgent to improve patients’ quality of life.

Green tea contains multiple polyphenolic compounds such as (−)-epigallocatechin-3-gallate (EGCG), (−)-epigallocatechin (EGC), (−)-epicatechin-3-gallate (ECG) and (−)-epicatechin (EC) (6). Among these, EGCG is the major active component and the most abundant catechin (7). EGCG has been intensively studied in the past years, mainly because of its diversified effects in chemoprophylaxis and disease treatment. Indeed, EGCG has been shown to possess anti-oxidative (8), antimicrobia (9), and anticarcinogenic (10) effects, in addition to reducing the risk for cardiovascular diseases (11), diabetes (12), neurodegenerative diseases (13) and cancer. The anticancer effects of EGCG on different cancer events have been assessed: EGCG reversed the progression of colon carcinomas by decreasing methylation (14), reduced mRNA expression and showed anti-proliferative potential in cervical cancer cells (15), induced apoptosis in skin cancer cells (16), and inhibited motility and angiogenesis of lung and breast cancer cells (17–19). In addition, the effect of EGCG on suppressing division and inducing apoptosis of cancer cells is mediated by several signaling pathways. EGCG has been shown to activate killer caspases and suppress NF-κB activation in epidermoid carcinoma A431 cells (20). It has also been suggested that EGCG induces apoptosis by activating caspases and MAPK pathways in lung adenocarcinoma and esophageal carcinoma cells (21). The PI3K/AKT/mTOR pathway is involved in EGCG-induced apoptosis of pancreatic carcinoma cells (22). Therefore, we hypothesized that EGCG may also induce BCL cells apoptosis.

Apoptosis is an essential process, which eliminates anomalous cells and balances the internal environment whose disturbance can bring about cancer (23). Intrinsic (mitochondrial) and extrinsic (death receptor) pathways are the major apoptosis routes. Apoptosis is controlled by a crossed pathway network which contains some targets for activity such as the crucial executor caspases (cysteine-dependent aspartate-specific proteases). The extrinsic pathway is triggered by tumour necrosis factor receptor (TNFR), Fas and TNF-related apoptosis-inducing ligand (TRAIL), and activates the initiator caspase-8. Subsequently, the signaling starts cascade reactions that activate caspase-3 and -7. The intrinsic pathway occurs when cytochrome *c* released from mitochondria to the cytosol acts with Apaf-1 to promote the activation of caspase-9 and the downstream members caspase-3 and -7 (24). Furthermore, B cell lymphoma-2 (Bcl-2) family members, including both pro-apoptotic (such as Bax and Bid) and anti-apoptotic (such as Bcl-2) molecules, modulate the mitochondrial pathway by mainly regulating the release of apoptosis factors such as cytochrome *c* (25).

Although EGCG was shown to be effective in multiple cancers, few studies have discussed the relationship between EGCG and lymphoma, especially MCL and BL. In this study, we assessed the effects of EGCG on Jeko-1 and Raji cells. We demonstrated that EGCG markedly suppresses cell growth. In addition, our results revealed that the EGCG-mediated growth inhibition occurs through increased activation of caspases and Bcl-2 family proteins that induce apoptosis. Consequently, EGCG may serve as emerging targeted therapy option for B cell lymphoma.

## Materials and methods

### Cell culture

The MCL Jeko-1 and BL Raji cell lines were kindly provided by Professor Hong Cen (Affiliated Tumour Hospital of Guangxi Medical University, Nanning, China). Cells were grown in RPMI-1640 medium supplemented with 10% fetal bovine serum (FBS, all from Hyclone, USA) for Raji cells and 20% FBS for Jeko-1 cells, in a humidified atmosphere containing 5% CO_2_ at 37°C. Cells were subcultured every 2–3 days.

### Cell proliferation assay

Cell growth inhibition effect was determined using Cell Counting Kit-8 (CCK-8) (Dojindo Co., Japan). Jeko-1 and Raji cells were plated at 4×10^4^ or 5×10^5^ cells/well in 96-well plates and cultured for 1–2 h. Then, cells were treated with different concentrations of EGCG (0–120 μg/ml) (Sigma-Aldrich, USA) for 12, 24 and 36 h. Cell culture medium of the same volume was used as control. After incubation, 10 μl CCK-8 reagent were added to each well and further incubated at 37°C for 2 h. Optical density (OD) values were measured at 450 nm with a microplate reader (Thermo Multiskan MK3, USA). Finally, growth inhibition rate (IR) was calculated as follows: IR (%) = (OD_control_−OD_experiment_)/(OD_control_−OD_backgroud_) ×100%.

### Flow cytometry analysis of apoptosis

Apoptosis was measured with Annexin V: PE apoptosis detection kit (BD Biosciences). First, Jeko-1 cells (5×10^5^ cells/well) or Raji cells (1×10^6^ cells/well) were plated in 6-well plates, respectively, and treated with different concentrations of EGCG (0, 20, 40 and 60 μg/ml) for 12, 24 and 36 h. Then, cells were washed with cold PBS and resuspended in 100 μl 1X binding buffer, followed by addition of 5 μl Annexin V-PE and 5 μl 7-AAD. The cells were incubated for 15 min at room temperature in the dark. Finally, 400 μl 1X binding buffer were added to the cells, which were analyzed by flow cytometry (BD Caliber, USA).

### RNA extraction and reverse transcription polymerase chain reaction (RT-PCR)

Jeko-1 and Raji cells were treated with EGCG (0, 20, 40 and 60 μg/ml) for 24 h. Total RNA was extracted using TRIzol reagent (Invitrogen, USA) according to the manufacturer’s instructions and quantified by NanoDrop2000 (Thermo Scientific, USA). Equal amounts of RNA (maximum 1 μg) were reverse-transcribed into cDNA using the ReverTra Ace^®^ qPCR RT kit (Toyobo, Japan). First, total RNA samples were incubated at 65°C for 5 min and kept on ice. Then, the denatured RNA was added to the reaction solution containing nuclease-free water, 5X RT buffer, RT enzyme mix and primer mix, and subsequently incubated at 37°C for 15 min and 98°C for 5 min. The resulting cDNA samples were stored at −20°C.

### Quantitative real-time PCR

Quantitative RT-PCR was performed in 20 μl reaction containing cDNA, nuclease-free water, primers and SYBR^®^ Green Real-time PCR Master Mix (Toyobo, Japan) according to the manufacturer’s instructions. Primers used were as follows: GAPDH, 5′-GTCAAGGCTGA GAACGGGAA-3′ (forward) and 5′-AAATGAGCCCCAGCCT TCTC-3′(reverse);Bcl-2,5′-AACATCGCCCTGTGGATGAC-3′ (forward) and 5′-AGAGTCTTCAGAGACAGCCAGGAG-3′ (reverse); Bax, 5′-AGATGTGGTCTATAATGCGTTTTCC-3′ (forward) and 5′-CAGAAGGCACTAATCAAGTCAAGGT-3′ (reverse); Fas, 5′-TCTGGTTCTTACGTCTGTTGC-3′ (forward) and 5′-CTGTGCAGTCCCTAGCTTTCC-3′ (reverse). The PCR was carried out for 30 sec at 95°C (pre-denaturation) followed by 35 cycles of 5 sec at 95°C (denaturation), 10 sec at 60°C (annealing) and 15 sec at 72°C (extension). GAPDH served as internal control to normalize the data. All treatments were performed in triplicate and gene expression was assessed by the 2^−ΔΔCt^ method.

### Analysis of caspase activity

Caspase-8 activation was assessed with the caspase colorimetric assay kit (KeyGen, China). This assay is based on spectrophotometric detection of the chromophore p-nitroanilide (pNA) released following the cleavage of the substrate caspase. Briefly, Jeko-1 or Raji cells were harvested after treatment with EGCG (0, 20, 40 and 60 μg/ml) for 24 h, and lysed on ice in 100 μl lysis buffer (containing 1 μl DTT) for 30 min. Cells lysates were centrifuged for 1 min at 10,000 × g and the supernatants assessed for protein content. Subsequently, supernatant samples containing 200 μg proteins were incubated with 50 μl reaction buffer (containing 0.5 μl DTT) and 5 μl caspase-8 substrate at 37°C for 4 h in the dark. The OD values were measured at 405 nm with a microplate reader. The activation level of caspase-8 was calculated by OD_experiment_/OD_control_.

### Western blot analysis

Jeko-1 or Raji cells were harvested after treatment with EGCG (0, 20, 40 and 60 μg/ml) for 24 h. To inhibit apoptosis, Z-VAD-FMK (Merck Millipore, Germany) was used to pretreat cells for 1 h before EGCG addition. Cell proteins were obtained after lysis on ice with RIPA lysis buffer [20 mM Tris (pH 7.5), 150 mM NaCl, 1 mM Na_2_EDTA, 1 mM EGTA, 1% Triton X-100, 2.5 mM sodium pyrophosphate, 1 mM β-glycerophosphate, 1 mM Na_3_VO_4_, 1 μg/ml leupeptin] (Cell Signaling Technology, USA) containing 1 mM PMSF. The lysates were clarified by centrifugation at 4°C for 10 min at 14,000 × g; protein content was measured by the BCA kit (Beyotime, China) according to the manufacturer’s instructions. Equal amounts of protein were mixed with loading buffer and preheated 5 min at 100°C. Proteins were electrophoretically separated on 10–15% sodium dodecyl sulfate (SDS)-polyacrylamide gels and transferred onto PVDF membranes on ice for 1–2 h. After blocking with 5% non-fat milk in TBST for 1 h, membranes were incubated with primary antibodies against β-actin, PARP, caspase-3, -7 and -9, cleaved proteins (PARP, caspase-3, -7 and -9), Bcl-2 and Bax overnight at 4°C. Then, the membranes were washed 3 times for 5 min with TBST and subsequently incubated with anti-rabbit IgG HRP-secondary antibody for 2 h and washed 3 times for 10 min with TBST. All antibodies were purchased from Cell Signaling Technology. Finally, immunoreactive proteins were detected with the ECL kit (Boster, China) and analyzed by GelDoc XR system (Bio-Rad, USA).

### Statistical analysis

All experiments were performed at least three times separately. The data are presented as mean ± SD. One-way ANOVA was used for comparisons among multiple groups. Statistical significance was established at p<0.05.

## Results

### EGCG inhibits proliferation of both Jeko-1 and Raji cells

**Figure 1:**
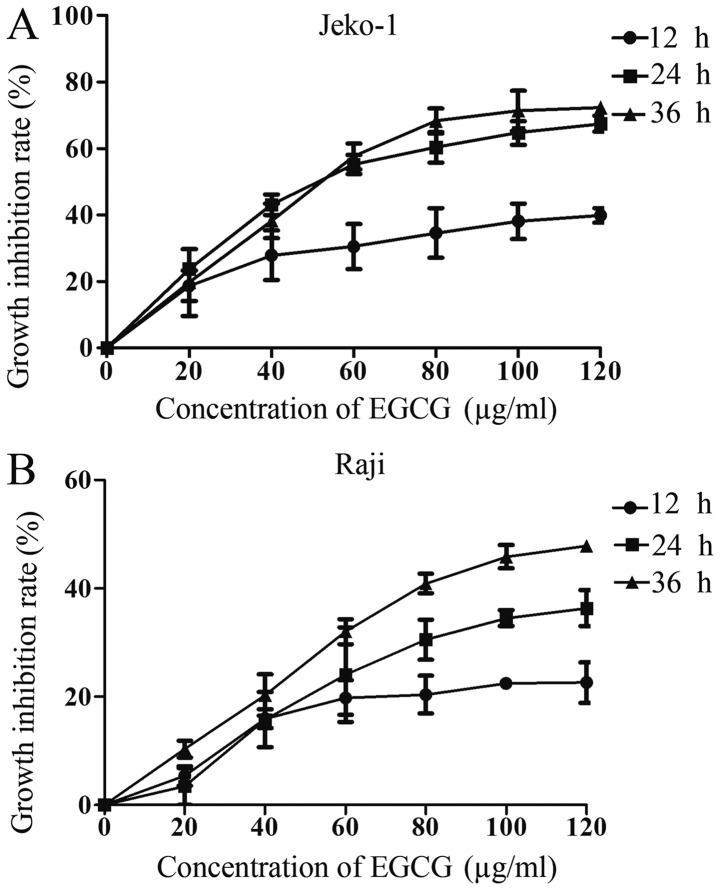
EGCG inhibits the proliferation of both Jeko-1 and Raji cells. Jeko-1 (A) or Raji (B) cells were treated with EGCG at different concentrations (0, 20, 40, 60, 80, 100 and 120 μg/ml) for 12, 24 and 36 h. The inhibition rates were determined by the CCK-8 assay. Results represent mean ± SD from three independent experiments.

To determine the effect of EGCG on cancer cells, Jeko-1 (Fig. 1A) or Raji (Fig. 1B) cells were treated with different EGCG concentrations and various time-points. The growth inhibition rate was assessed by the CCK-8 assay. As demonstrated in Fig. 1, cell inhibition rate at various time-points increased with EGCG concentration in both cell lines. The half-maximal inhibitory concentration (IC_50_) of EGCG at 36 h for Jeko-1 and Raji was 57.98 and 61.24 μg/ml, respectively. Therefore, 20, 40 and 60 μg/ml were selected for subsequent experiments. These data indicated a time- and dose-dependent effect of EGCG on growth inhibition of both Jeko-1 and Raji cells.

### EGCG induces apoptosis in Jeko-1 and Raji cells

**Figure 2:**
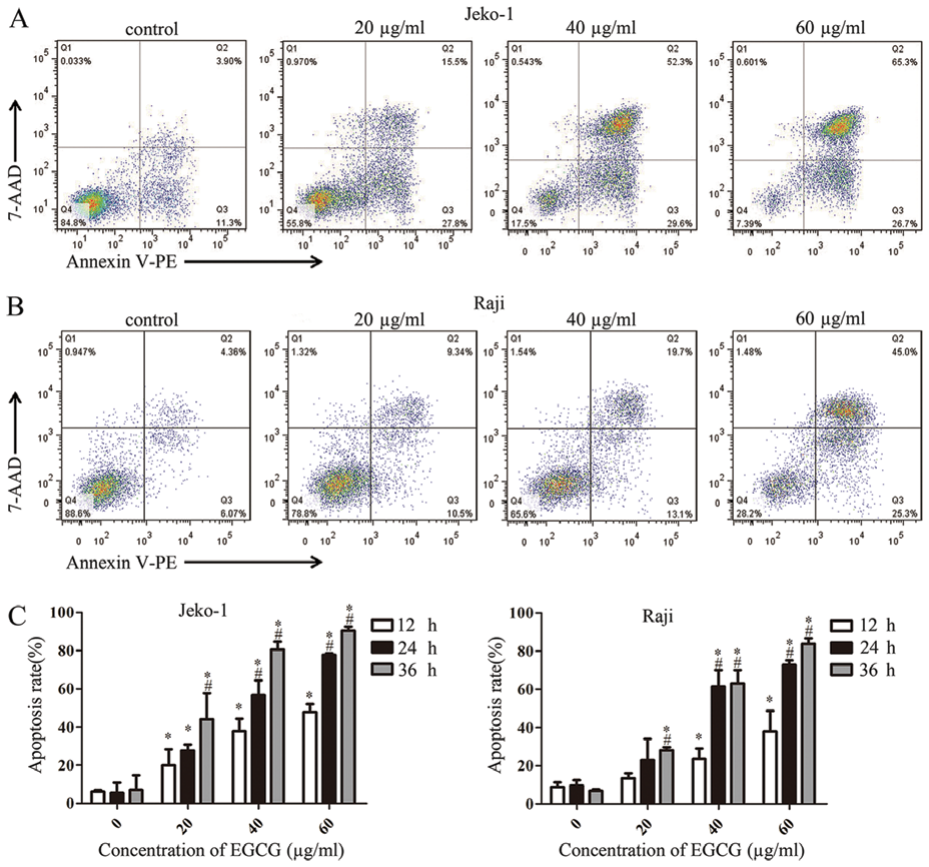
EGCG induces apoptosis in Jeko-1 and Raji cells. Jeko-1 (A) or Raji (B) cells were treated with EGCG (0, 20, 40 and 60 μg/ml) for 24 h and stained with Annexin V-PE/7-AAD before flow cytometry analysis. Representative results from three independent experiments are shown. (C) Jeko-1 or Raji cells were treated with EGCG at different concentrations (0, 20, 40 and 60 μg/ml) for 12, 24 and 36 h. Data represent mean ± SD from three independent experiments. ^*^p<0.05, compared with the control group for the same time-point. ^#^p<0.05, compared with 12 h for the same concentration.

To assess whether EGCG-associated growth inhibition resulted from induced apoptosis in Jeko-1 and Raji cells, Annexin V-PE and 7-AAD were used to stain cells treated with different EGCG concentrations. Annexin V is a protein with high affinity for the phospholipid phosphatidylserine (PS) exposed from inner layer to external environment in apoptotic cells; thus, Annexin V is used to determine undergoing apoptosis. 7-Amino-actinomycin (7-AAD) is a probe used to distinguish viable cells from dead and damaged ones. As shown in Fig. 2, apoptotic Jeko-1 (Fig. 2A) and Raji (Fig. 2B) cells in Q2 (early apoptosis) and Q3 (late apoptosis) increased gradually with EGCG concentrations. Fig. 2C shows that apoptosis rates in Jeko-1 and Raji cells were time- and dose-dependent, in agreement with CCK-8 assay data.

### EGCG increases the activities of caspase-3, -7 and PARP

**Figure 3:**
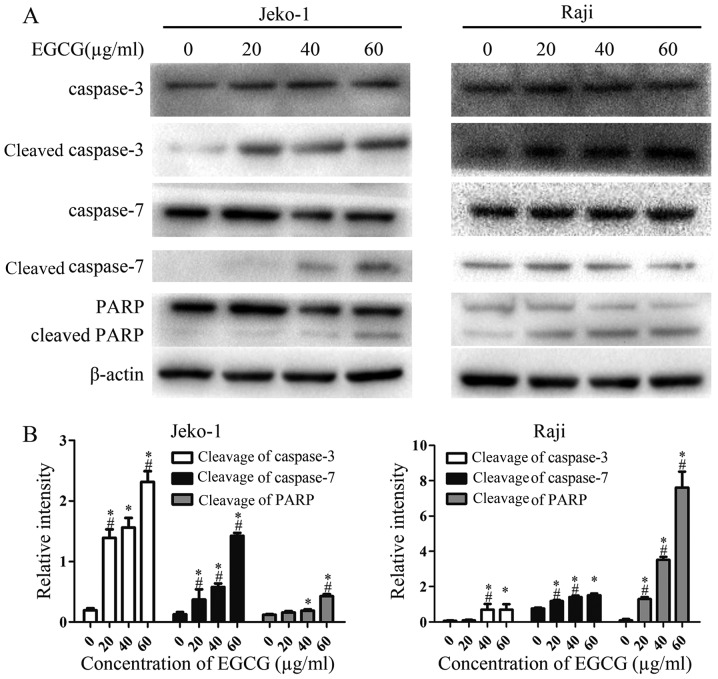
EGCG increases the activation of caspase-3, -7 and PARP. (A) Jeko-1 or Raji cells were treated with EGCG at different concentrations (0, 20, 40 and 60 μg/ml) for 24 h. Equal amounts of total protein were examined by western blot analysis with indicated antibodies. β-actin was used as a loading control. Representative data from three independent experiments are shown. (B) Relative intensity of cleaved caspase-3, -7 and PARP, normalized by uncleaved caspase-3, -7 and PARP. Values represent mean ± SD from three independent experiments. ^*^p<0.05, compared with control cells (0 μg/ml).

To further investigate the mechanisms involved in EGCG-induced apoptosis, the activities of caspase-3, -7 and PARP were determined by western blot analysis. As shown in Fig. 3, EGCG significantly increased caspase-3, -7 and PARP activities, and activation levels increased in a dose-dependent manner. This suggests a relationship between caspase activity and the EGCG-induced apoptosis in Jeko-1 and Raji cells.

### Effect of the general caspase inhibitor Z-VAD-FMK on EGCG-induced activity of caspase-3, -7 and PARP

To determine the role of caspases in EGCG-induced apoptosis, cells were pretreated with Z-VAD-FMK. As expected, Z-VAD-FMK sufficiently inhibited the EGCG-induced activities of caspase-3, -7 and PARP. Taken together, the above data strongly suggested that the EGCG-induced apoptosis rely on caspase-dependent pathways in Jeko-1 and Raji cells.

### EGCG induces apoptosis through the death receptor pathway: increases caspase-8 activity and Fas upregulation

To explore how the upstream molecules triggered the apoptosis executors caspase-3 and -7, and their substrate PARP, we first determined caspase-8 activity using the caspase colorimetric assay kit. As shown in Fig. 5A EGCG-treated cells showed a dose-dependent increase of caspase-8 activity. Then, to explore the upstream effector that actives caspase-8, the expression of Fas mRNA was assessed by RT-PCR. Fig. 5B showed that Fas mRNA expression levels increased in a dose-dependent manner in EGCG-treated cells. Of note, the maximum caspase-8 activation and Fas mRNA expression levels in Jeko-1 cells were at least doubled compared with controls and significantly higher than in Raji cells. These results suggested that EGCG-induced apoptosis was associated with the death receptor pathway.

### EGCG affects Bcl-2 and Bax mRNA expression

In order to investigate whether other molecular events were involved in EGCG-induced apoptosis in Jeko-1 and Raji cells, we examined the expression levels of Bcl-2 and Bax mRNA by RT-PCR. As shown in Fig. 6, EGCG upregulated Bax mRNA expression in a dose-dependent manner; in contrast, Bcl-2 was downregulated, also in a dose-dependent manner. These results indicated that Bcl-2 and Bax were also involved in EGCG-induced apoptosis.

### EGCG induces apoptosis through the mitochondrial pathway: increases caspase-9 activity and regulation of Bcl-2 and Bax

In order to further confirm the involvement of the mitochondrial pathway, the most important and common apoptosis pathway, in EGCG-induced apoptosis in Jeko-1 and Raji cells, caspase-9 activity and expression levels of Bcl-2 family proteins Bcl-2 and Bax were assessed. Treatment of both cell lines with EGCG resulted in increased activity of caspase-9 (Fig. 7A and B). The expression of the anti-apoptotic protein Bcl-2 was downregulated significantly, whereas that of the pro-apoptotic protein Bax was upregulated. Importantly, these changes were also EGCG dose-dependent. When cells were pretreated with the general caspase inhibitor Z-VAD-FMK, caspase-9 activity was decreased compared with the EGCG-treated group as indicated in Fig. 7C and D. These results suggested that EGCG triggers the mitochondrial pathway, regulating Bcl-2 family proteins to induce apoptosis in Jeko-1 and Raji cells.

## Discussion

Natural substances are increasingly used around the world for both chemoprophylaxis and treatment of various diseases. For instance, EGCG has attracted growing attention due to its low toxicity in normal cells and significant inhibitory effect on cancer cells (26,27). Our previous research demonstrated that EGCG significantly inhibits the growth of hepatocellular carcinoma cells SMMC7721 at 48 h (28). In this study, we assessed the apoptotic effects of EGCG in B lymphoma cells and investigated the possible molecular mechanisms. We showed that EGCG significantly inhibits Jeko-1 and Raji cell growth in a dose- and time-dependent manner (Fig. 1), in accordance with previous studies assessing other cancer cells (16,26,27).

Apoptosis is an essential process and fundamental cellular activity, which eliminates anomalous cells. Inducing cancer cell apoptosis is the key to cancer treatment, and recent studies have shown that many natural substances induce apoptosis as their primary anticancer mechanism (29–31). Therefore, apoptosis rate is considered an indicator of anticancer activity. As shown above (Fig. 2), the apoptotic cell rate increased significantly with time and EGCG dose. In agreement, Hazawa *et al* suggested that EGCG causes time-dependent apoptosis in Raji cells (32). These findings indicated that EGCG inhibits cell growth through induction of apoptosis.

**Figure 4:**
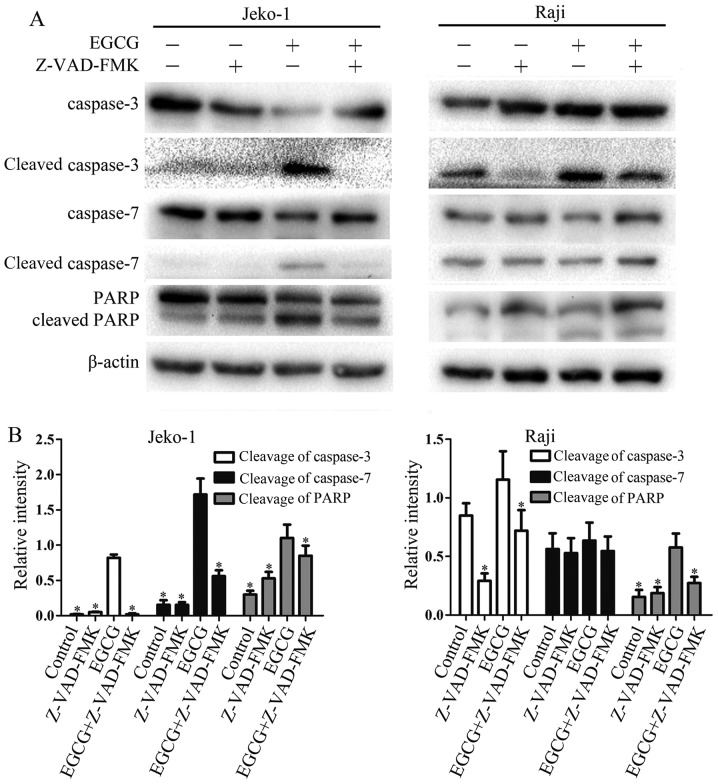
Z-VAD-FMK reverses the EGCG-induced activation of caspase-3, -7 and PARP. (A) Jeko-1 or Raji cells were pretreated with Z-VAD-FMK (10 μM) for 1 h and incubated with 60 μg/ml EGCG for 24 h. Equal amounts of total protein were examined by western blot analysis using the indicated antibodies. β-actin was used as a loading control. (B) Relative intensity of activated caspase-3, -7 and PARP. The values represent mean ± SD from three times independent experiments. ^*^p<0.05, compared with the EGCG-treatment group.

Previous studies have proposed that potential novel agents should mainly inhibit growth and induce apoptosis in different lymphoma cells. Activation of caspases is a central process of the 2 major apoptosis pathways, and activated caspases provide a link between cell signaling and apoptotic execution (24). It has been reported that green tea polyphenols induce cell death by caspase-3 activation (33). We investigated the molecular mechanisms of EGCG induced Jeko-1 and Raji cells growth inhibition, and found a dose-dependent increase of caspase-3 and -7 activation (Fig. 3). PARP is associated with DNA damage in apoptosis and is the major downstream substrate of caspase-3 (34). PARP activation increased overtly with EGCG concentration as shown in Fig. 3. Furthermore, the activation of caspase-3, -7 and PARP was inhibited by the general caspase inhibitor Z-VAD-FMK (Fig. 4). These results indicated that EGCG-induced apoptosis is caspase-dependent in Jeko-1 and Raji cells. This finding was consistent with a previous study demonstrating that EGCG suppresses VEGF-R phosphorylation and induces apoptosis by increasing the activity of caspase-3 and PARP in chronic lymphocytic leukemia B cells (35).

**Figure 5:**
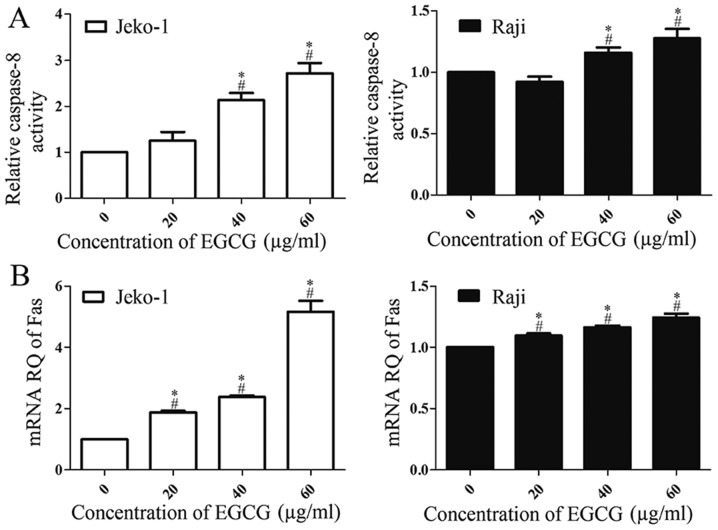
EGCG increases the activation of caspase-8 and upregulates Fas mRNA levels. (A) Jeko-1 or Raji cells were treated with EGCG at different concentrations (0, 20, 40 and 60 μg/ml) for 24 h. Equal amounts of total protein were analyzed by the caspase-8 colorimetric assay kit, and the activation level is presented by OD_experiment_/OD_control_. (B) The cells were treated as in (A), and Fas mRNA expression was measured by RT-PCR. The relative quantification (RQ) was normalized to GAPDH levels. The values represent mean ± SD from three independent experiments. ^*^p<0.05, compared with the control group (0 μg/ml). ^#^p<0.05, compared with the previous group.

Next, we focused on upstream molecules to figure out which pathway triggers the EGCG-induced apoptosis. The death receptor pathway triggered by stimulation of the death receptor CD95 (APO-1/Fas) results in receptor aggregation, and FADD and caspase-8 recruitment; subsequently, caspase-8 becomes activated and directly leads to a cascade that activates the apoptosis executor caspase-3 (36). The role of EGCG and a radiolytic product in triggering the Fas-caspase-8-medicated pathway in lymphoma U937 cells has been demonstrated by others (37,38). The enhanced expression of Fas mRNA and cleavage of caspase-8 with increasing EGCG concentrations observed in this study demonstrated that EGCG initiates the death receptor pathway to induce apoptosis (Fig. 5), in accordance with the above previous studies.

**Figure 6:**
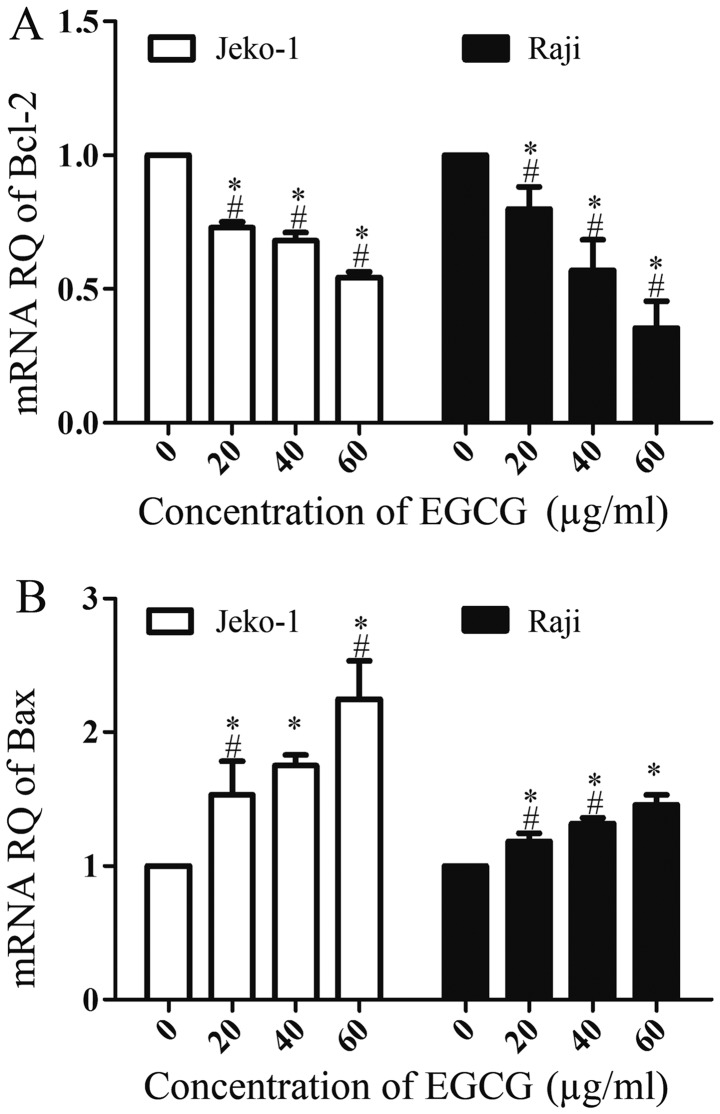
EGCG upregulates Bcl-2 and Bax transcription. (A and B) Jeko-1 or Raji cells were treated with EGCG at different concentrations (0, 20, 40 and 60 μg/ml) for 24 h and total RNA was extracted. The transcription levels of Bcl-2 and Bax were measured by RT-PCR. The relative quantification (RQ) was normalized to GAPDH levels. Data represent mean ± SD from three independent experiments. ^*^p<0.05, compared with the control group (0 μg/ml). ^#^p<0.05, compared with the previous group.

**Figure 7:**
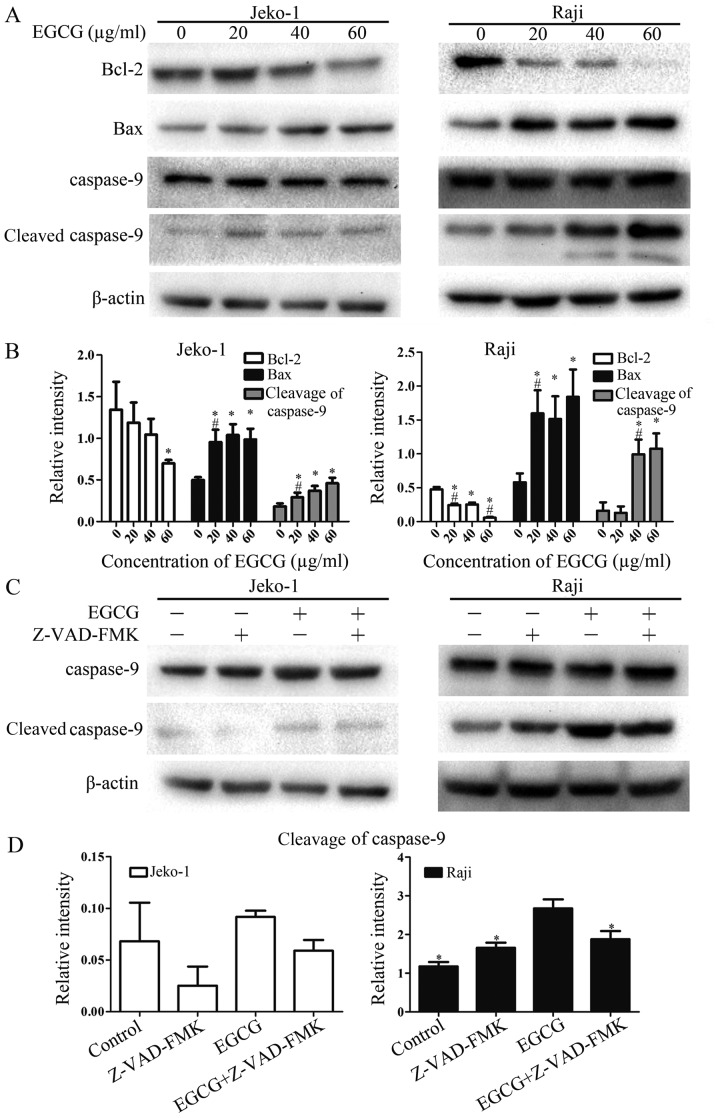
EGCG upregulates Bcl-2 and Bax expression, and caspase-9 activation. (A) Jeko-1 or Raji cells were treated with EGCG at different concentrations (0, 20, 40 and 60 μg/ml) for 24 h. Equal amounts of total protein were examined by western blot analysis, with appropriate antibodies. β-actin was used as a loading control. (B) Relative band intensities of Bcl-2, Bax and activated caspase-9. (C) The cells were pretreated with the general caspase inhibitor Z-VAD-FMK (10 μM) for 1 h and incubated with EGCG (60 μg/ml) for 24 h. Equal amounts of total protein were examined by western blot analysis to determine the inhibition of EGCG-induced caspase-9 activation. ^*^p<0.05, compared with the control group (0 μg/ml); ^#^p<0.05, compared with the previous group. (D) Inhibition of caspase-9 activation shown by relative band intensity detected by western blot analysis using appropriate antibodies. β-actin was used as a loading control. The values represent mean ± SD from three independent experiments. ^*^p<0.05, compared with the EGCG-treatment group.

The mitochondrial pathway has been shown by multiple studies to play a major role in malignant cell apoptosis induced by many natural agents, including EGCG (25–27). The Bcl-2 family plays a pivotal role in regulating cell life and death; many of these apoptosis-related proteins reside in the outer mitochondrial membrane. Once the balance of Bcl-2 (preventing cell survival from various chemotherapeutic agents) and Bax (a pro-apoptosis protein) is broken, the proteins move towards the cytosol and modulate mitochondria to promote cytochrome *c* release, then combine with caspase-9 to form a complex that triggers caspase-3 activation (39,40). Our results also indicated that EGCG increases the protein and mRNA expression of Bax as well as caspase-9 activation while decreasing the expression of Bcl-2, all in a dose-dependent manner (Figs. 6 and 7). In other words, EGCG initiated the mitochondrial pathway at the same time to induce apoptosis in Jeko-1 and Raji cells. However, it has been demonstrated that EGCG induces apoptosis in Hep2 cells, relying on p53-mediated and AIF-dependent mitochondrial pathway, which is not a caspase-dependent pathway (41). In addition, EGCG was shown to induce apoptosis in imatinib-resistance chronic myelogenous leukemia K562 cells via a caspase-independent mechanism (42). Whether this phenomenon is idiosyncratic requires further studies in other cell lines. Previous studies have revealed the means by which EGCG or other natural agents cause the mitochondria to release cytochrome *c*: one direct means is mediated by Bcl-2 family proteins; the other is through Fas aggregated caspase-8, not directly activating the caspase-3 cascade but followed by cleaving of Bid to tBid and subsequent cytochrome *c* release that activates caspase-9 and caspase-3 (37,43). Although our results suggested that EGCG induces apoptosis through both the extrinsic (death receptor) and intrinsic (mitochondrial) pathways, it is unclear whether the two pathways are linked by Bid cleavage in Jeko-1 and Raji cells. To our knowledge, the present data demonstrate for the first time that EGCG induces cytotoxicity in MCL Jeko-1 cells. The cytotoxic effect of EGCG on BL Raji cells corroborates with a previous study (32). We further unveiled the molecular mechanisms by which EGCG activates the caspase pathways to cause apoptosis of MCL and BL cells. Our results indicate that EGCG-induced apoptosis mainly involves both the death receptor and mitochondrial pathways, which depend on the activation of caspase-3, -7, -8, -9 and PARP as well as the mediation of Bcl-2 family proteins. Overall, EGCG may be a potential novel therapeutic agent against B cell lymphoma.
