# [Corrigendum] (-)-Epigallocatechingallate induces apoptosis in B lymphoma cells via caspase-dependent pathway and Bcl-2 family protein modulation

**DOI:** 10.3892/ijo.2024.5683

**Published:** 2024-08-28

**Authors:** Jiangyan Wang, Yu'an Xie, Yan Feng, Litu Zhang, Xinping Huang, Xiaoyun Shen, Xiaoling Luo

Int J Oncol 46: 1507-1515, 2015; DOI: 10.3892/ijo.2015.2869

Subsequently to the publication of the above article, an interested reader drew to the authors' attention that, in Fig. 3 on p. 1510, the western blot images selected to portray the caspase 7 and PARP/cleaved PARP experiments were remarkably similar.

After having referred to their original data, the authors realized that the PARP/cleaved PARP blots had been inadvertently duplicated in the figure. The revised version of Fig. 3, showing the correct data for the caspase-7 experiment, is shown below. The authors confirm that the errors made during the assembly of Fig. 3 did not adversely affect the major conclusions presented in this paper, and are grateful to the Editor of *International Journal of Oncology* for allowing them this opportunity to publish a corrigendum. They also apologize to the readership for any inconvenience caused.

## Figures and Tables

**Figure 3:**
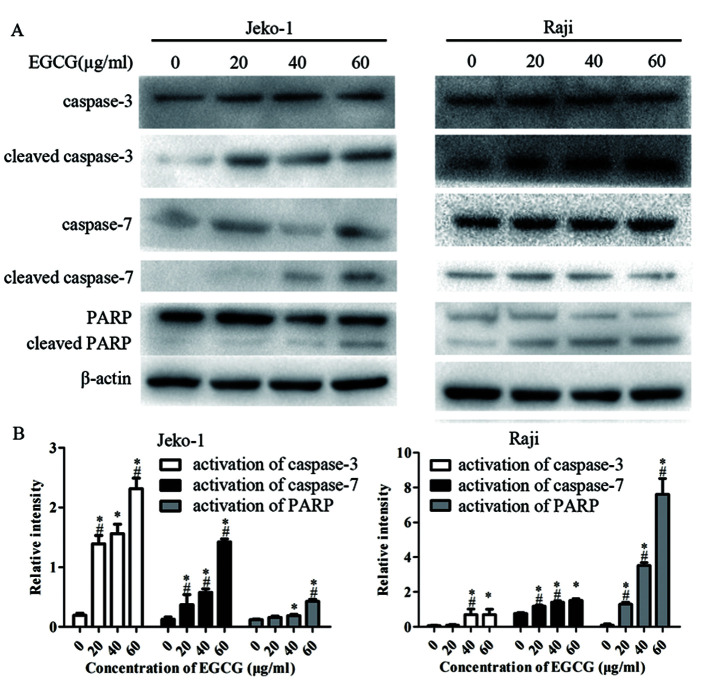
EGCG increases the activation of caspase-3, -7 and PARP. (A) Jeko-1 or Raji cells were treated with EGCG at different concentrations (0, 20, 40 and 60 μg/ml) for 24 h. Equal amounts of total protein were examined by western blot analysis with indicated antibodies. β-actin was used as a loading control. Representative data from three independent experiments are shown. (B) Relative intensity of cleaved caspase-3, -7 and PARP, normalized by uncleaved caspase-3, -7 and PARP. Values represent mean ± SD from three independent experiments. ^*^p<0.05, compared with control cells (0 μg/ml).

